# Rescue of Non-Informative Circulating Tumor DNA to Monitor the Mutational Landscape in NSCLC

**DOI:** 10.3390/cancers12071917

**Published:** 2020-07-16

**Authors:** Stefanie Mayer, Gerlinde Schmidtke-Schrezenmeier, Christian Buske, Frank G. Rücker, Thomas F.E. Barth, Peter Möller, Ralf Marienfeld

**Affiliations:** 1 Institute of Pathology, University Medical Center Ulm, 89070 Ulm, Germany; stefanie.mayer@uniklinik-ulm.de (S.M.); thomas.barth@uniklinik-ulm.de (T.F.E.B.); peter.moeller@uniklinik-ulm.de (P.M.); 2 Department of Internal Medicine II, University Medical Center Ulm, 89070 Ulm, Germany; gerlinde.schmidtke-schrezenmeier@uniklinik-ulm.de; 3 Institute of Experimental Tumor Research, University Medical Center Ulm, 89070 Ulm, Germany; christian.buske@uni-ulm.de; 4 Department of Internal Medicine III, University Medical Center Ulm, 89070 Ulm, Germany; frank.ruecker@uniklinik-ulm.de

**Keywords:** liquid biopsy, cell-free DNA, NGS, precision medicine, targeted therapy

## Abstract

In non-small cell lung cancer (NSCLC) the usage of plasma-derived circulating tumor DNA (ctDNA) have come into focus to obtain a comprehensive genetic profile of a given lung cancer. Despite the usage of specific sampling tubes, archived plasma samples as well as inappropriately treated blood samples still cause a loss of information due to cell lysis and contamination with cellular DNA. Our aim was to establish a reliable protocol to rescue ctDNA from such non-informative samples to monitor the mutational landscape in NSCLC. As a proof-of-concept study we used archived plasma samples derived from whole blood EDTA samples of 51 patients suffering from NSCLC. Analysis of the isolated plasma DNA determined only a small fraction of ctDNA in a range of 90–250 bp. By applying a specific purification procedure, we were able to increase the informative ctDNA content and improve in a cohort of 42 patients the detection of driver mutations from 32% to 79% of the mutations found in tissue biopsies. Thus, we present here an easy to perform, time and cost effective procedure to rescue non-informative ctDNA samples, which is sufficient to detect oncogenic mutations in NGS approaches and is therefore a valuable technical improvement for laboratories handling liquid biopsy samples.

## 1. Introduction

Lung cancer is the leading cause in cancer related deaths throughout the world, with non-small cell lung cancer (NSCLC) being the most common subtype [1,2]. However, despite improvements in diagnosis and treatment, the 5-year survival rate remains dismal, due to late diagnosis, metastases and poor responsiveness to chemotherapy [3]. Therefore, early detection methods as well as personalized treatment using small molecule inhibitors on the basis of genetic profiling of a tumor sample are of high importance. During the last years several oncogenic mutations and genetic rearrangements have been discovered in NSCLC, with *EGFR, KRAS, ALK, MET, BRAF, PIK3CA, ROS1, HER2* and *RET* being the most abundant alterations in NSCLC adenocarcinoma [4]. Some of these oncogenic mutations, e.g., EGFR L858R, can be targeted with specific tyrosine kinase inhibitors [5]. However, tumor heterogeneity is common in NSCLC samples, and mutational profiles vary between different metastatic sites or even between different subclones in one site leading to an intra-individual heterogeneity in their genetic profile [6]. Thus, profiling a single tissue biopsy snapshot might be insufficient to unravel the tumor heterogeneity, potentially leading to false negative results [7]. For instance, a driver mutation located in an underrepresented subclone might be missed thus excluding a therapeutic option leading to treatment failure [1]. As the analysis of multiple biopsies appears to determine the complete tumor mutational landscape of NSCLC is not feasible, the usage of circulating tumor DNA isolated from blood plasma came into focus. Sozzi et al. demonstrated that plasma DNA concentration is increased in cancer patients compared to healthy donors, suggesting that an increase in the plasma DNA amount is an early event in lung carcinogenesis [8]. Likewise, in breast cancer higher plasma DNA levels were observed, despite inter-individual variations [9]. Hence, analysis of circulating tumor DNA (ctDNA) as a non-invasive tool is extensively studied. However, ctDNA is only defined by its molecular size of 90–250 bp and includes a minor amount of small fragment cell-free DNA from normal cells, but consists of a majority of small fragment cell-free DNA from tumor cells [10]. This technique is thought to provide an insight into the tumor heterogeneity, allowing one to monitor the mutational evolution during treatment, and offering a chance for molecular genotyping in case sufficient tissue material is not available. Therefore, ctDNA is thought to be an alternative surrogate for molecular analysis in cancer patients, requiring only a small amount of blood [11]. For instance, ctDNA from late stage NSCLC patients is used to test for additional EGFR mutations leading to resistance against EGFR tyrosine kinase inhibitors [12]. Of note, there are several limitations for the use of plasma samples. It is recommended to perform plasma isolation soon after blood withdrawal, while inappropriate handling and storage leads to non-informative liquid biopsy samples due to leukocyte lysis and therefore contamination with genomic DNA [13,14,15], thereby leading to non-suitable therapeutic options and treatment failure of those inappropriately handled samples [16]. Further, the usage of different blood collection tubes and centrifugation protocols for plasma isolation might cause a contamination of the plasma samples with high molecular genomic DNA to variable degrees [17,18,19,20]. However, how to use those non-appropriate handled liquid biopsy samples for further NGS approaches and how to rescue ctDNA from those samples remains a matter of debate.

As the contamination with genomic DNA is a main issue for daily routine lab work in molecular pathology as well as for multi-center studies including archived plasma samples, our aim was to establish a protocol to rescue non-informative DNA samples derived from inappropriately handled liquid biopsy samples and evaluate the feasibility of NGS approaches.

## 2. Results

### 2.1. Differences in Sample Handling Leading to Contamination with Cellular DNA

To analyze the applicability of archived plasma samples for mutational testing, a total of 51 patients suffering from NSCLC of the histological subtype adenocarcinoma and squamous cell carcinoma were included in a proof-of-concept study. Baseline characteristics of this patient cohort are summarized in Table 1.

**Figure 1:**
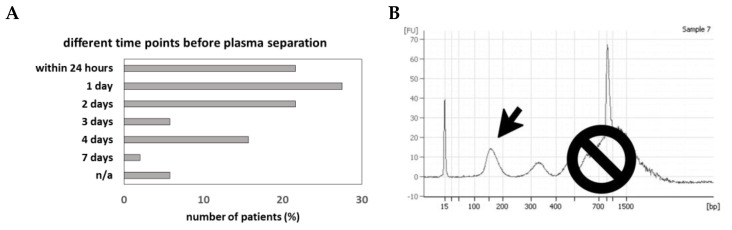
Delay in plasma separation. Fifty-one patients were enrolled in the study of which plasma preparation was performed at different time points after blood withdrawal. For all samples plasma DNA was isolated. (**A**) Storage time of whole blood in K_2_EDTA tubes at room temperature before plasma isolation. (**B**) Contamination of ctDNA (arrow) with high molecular genomic DNA (prohibition sign) was analyzed using an Agilent Bio-Analyzer.

As blood sampling and plasma preparation was performed before liquid biopsy analysis was part of the routine laboratory setup, the regulations regarding processing, shipping and storage time of the blood samples prior to plasma preparation was less well defined. Hence, duration to plasma preparation after blood withdrawal was variable spanning from a few hours to several days at room temperature. For the majority of the samples plasma isolation was performed 1–2 days after blood was drawn (49%, *n* = 25), whereas for eleven patients (21%) plasma was isolated within 24 h and for one patient plasma isolation was performed 7 days after blood withdrawal (Figure 1A).

**Figure 2:**
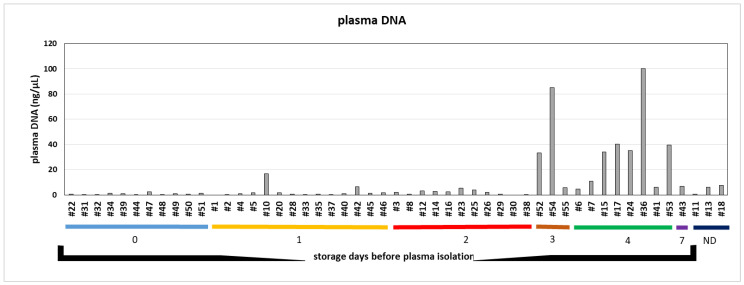
Relevance of time delay for total plasma DNA concentration. Plasma DNA concentrations of plasma samples isolated at different timepoints after venipuncture. Samples#9, #19, #21 and #27 were not processed due to a lack of material (ND = not documented).

High inter-individual variations in plasma DNA concentration were observed ranging from non-detectable plasma DNA to 100 ng/µL with a mean concentration of 9.52 ng/µL independent of storage time before plasma isolation (Figure 2). However, mean plasma DNA concentration of samples stored 4 days at room temperature before plasma isolation was significant higher compared to those stored 2 days or less at room temperature before plasma has been isolated (*p* < 0.0001, Figure 2). These data underline the impact of delay in sample processing on plasma DNA content.

**Figure 3:**
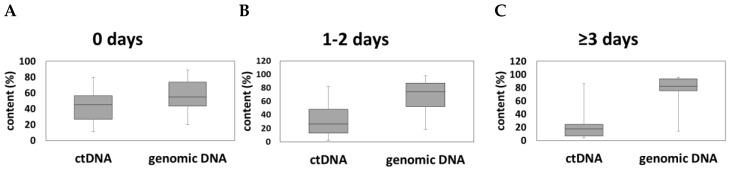
Delay in plasma preparation leads to increased plasma DNA contamination. CtDNA content and content of genomic DNA from plasma DNA samples isolated (**A**) at the same day, (**B**) at 1–2 days and (**C**) at ≥3 days after venipuncture.

To monitor the size distribution of the plasma DNA and therefore define the degree of contamination with high molecular cellular DNA, we analyzed the fragment size of the extracted plasma DNA using a Bio-Analyzer 2100. Circulating tumor DNA (ctDNA) has a fragment size of 90–250bp, while high-molecular cellular DNA, probably from lysed immune cells, are high molecular fragments clustering at the length of ≥1000 bp [21]. Fragment size analysis suggested that only a minor fraction of the isolated plasma DNA was in the range expected for ctDNA (Figure 1B). All samples contained longer DNA fragments ranging from 14% to 99% with a mean of 69%, suggesting a contamination with high molecular cellular DNA. We subdivided our samples into three subgroups—(1) plasma isolation within 24 h after blood withdrawal, (2) plasma isolation between 1 and 2 days after blood withdrawal and (3) plasma isolation at least 3 days after blood withdrawal. The percentage of isolated ctDNA representing a fragment region of 90–250 bp was decreased with prolonged time between blood withdrawal and plasma isolation, with a mean of 42% ctDNA for plasma samples handled the same day after blood withdrawal and 23% ctDNA for samples for which plasma isolation was performed at least 3 days after blood withdrawal (Figure 3A–C). Thus, the content of longer DNA fragments was slightly increased from 58% to 77% when plasma isolation was performed 3 days after venipuncture compared to those isolated within 24 h (Figure 3A–C). However, despite a larger variation between samples within the same subgroup in regard of their ctDNA content, we observed a significant difference between these two groups (*p* = 0.042).

### 2.2. Size Selection Based Purification of CtDNA from Archived Historic Plasma Samples

**Figure 4:**
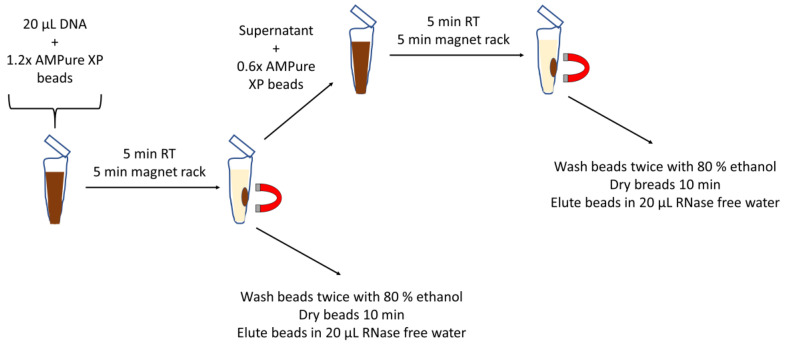
Purification procedure for the isolation of ctDNA from contaminated plasma DNA samples. Contaminated plasma DNA was mixed with AMPure XP magnetic beads, incubated and high molecular DNA bound to the magnetic beads were isolated. The pellet containing the high molecular DNA was washed twice with 80% ethanol, air-dried and the DNA was eluted with 20 µL RNase free water. Magnetic beads were added to the supernatant (0.6× volume of supernatant), incubated, pelleted on a magnetic rack. Washing, drying and elution of the ctDNA was done similarly.

The high degree of contaminating high-molecular DNA might affect the mutational profiling of the ctDNA probably resulting in false negative sequencing results. In order to rescue the contaminated plasma DNA samples, we established a size-selection protocol for the purification of the ctDNA (Figure 4). 

**Figure 5:**
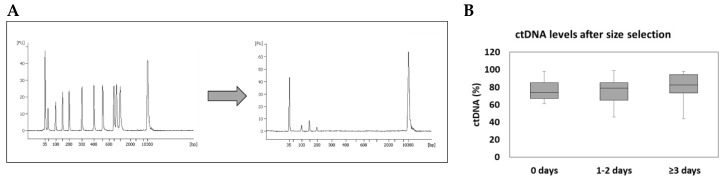
CtDNA levels in rescued plasma DNA samples. (**A**) Using the size selection protocol small fragments in a range of 90–250 bp from the Agilent high sensitivity DNA ladder were selectively separated. (**B**) Successful purification from all 51 plasma samples was independent of storage time before plasma isolation.

With this protocol we selectively purified DNA fragments with a size of 90–250 bp from the Agilent DNA 1000 DNA ladder used as control (Figure 5A). To explore whether the purification procedure is able to rescue non-informative plasma DNA sample for a further analysis of known driver mutants, we processed 51 archived liquid biopsy samples using the purification procedure and determined its efficacy by an analysis with the Agilent Bio-Analyzer 2100. 

**Figure 6:**
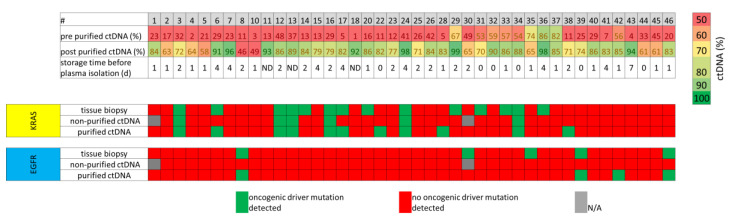
Improved sensitivity of oncogenic gene mutations in rescued plasma DNA samples. CtDNA content pre and post purification as well as duration to plasma preparation was shown for each sample. DNA was purified up to 44–99% with a mean of 77% ctDNA. Comparison of resequencing analyses using tumor tissue samples, non-purified plasma DNA samples (pre purified ctDNA) and purified DNA samples (post purified ctDNA) of 42 patients with non-small cell lung cancer (NSCLC) with an eight-gene panel including *EGFR, KRAS, IDH1, IDH2, PDGFRA, NRAS, BRAF* and *KIT*. Samples with oncogenic mutations in *KRAS* (middle part) or *EGFR* (lower part) are labeled green, all samples without driver mutations were labeled red (ND = not documented; N/A = not available).

The plasma DNA samples were significantly purified up to 99% ctDNA with a mean of 77% ctDNA compared to 31% ctDNA of the non-purified samples (*p* < 0.0001, Figure 5B and Figure 6). Moreover, purification was independent of the storage time before plasma isolation (Figure 5B) and the degree of contamination with higher molecular DNA before size selection procedure (Figure 6, Table S1). For example, plasma preparation of sample #43 was done 7 days after venipuncture and ctDNA content was 4% prior purification. However, after size selection ctDNA content was 94% (Figure 6). Out of 51 samples only for four samples we were unable to reach a purity of 50%, while for 47 samples we reached purity of 58% and higher (Figure 6, Table S1). In addition, purification efficacy was independent of the UICC7 stage, as we obtained purification of 60% for patients with NSCLC stage IA, 78% for patients with NSCLC stage IIA/B, 87% for patients with NSCLC stage IIIA/B and 76% for patients with NSCLC stage IV.

In case the purification was incomplete, this process can be repeated as often as needed until the best purity with a minimal loss of ctDNA is achieved. Moreover, to avoid a loss of the ctDNA fraction during the purification step, the high molecular DNA fraction was also eluted and monitored for remaining ctDNA. In this case, we did a second purification procedure using the eluate from the beads to avoid loss of ctDNA. In total, mean reduction between pre-purified and post-purified ctDNA was 11 ng/mL plasma, ranging from 0 to 123 ng/mL plasma (Table S2). After the purification procedure there was no significant difference between samples of different subgroups (Figure 5B).

### 2.3. Sample Purification Rescues Non-Informative Plasma DNA Samples for Mutational Analysis

In order to determine the impact of the purification procedure on the usability of the plasma DNA samples for a mutational analysis, we subjected 42 purified ctDNA samples as well as their non-purified counterparts of which sequencing data from the corresponding tissue material was available to a targeted resequencing approach with an eight gene panel including *BRAF, EGFR, IDH1, IDH2, KIT, KRAS, NRAS* and *PDGFRA* used for molecular diagnostics in our routine laboratory work. The results were compared with the mutation status obtained with the corresponding 42 tissue samples. We also included the two purified ctDNA samples, which did not reach the 50% ctDNA purity (#8 and #10), while the non-purified samples #1 and #30 were excluded due to insufficient DNA amounts.

**Figure 7:**
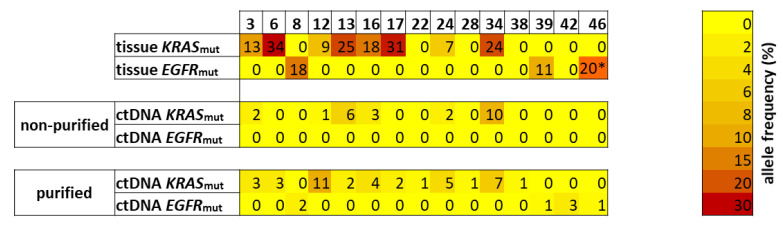
Decreased allele frequency in non-purified ctDNA samples. Heatmap showing the variant allele frequency (%) of detected oncogenic mutations in purified and non-purified ctDNA in comparison to the detected mutations using tissue biopsies (*estimated from Sanger sequencing).

Analysis of the tissue biopsy samples revealed 23 samples without detectable oncogenic mutation, five samples with an oncogenic *EGFR* mutation and 14 samples with an oncogenic *KRAS* mutation. None of the oncogenic *EGFR* mutations and only six of the 14 oncogenic *KRAS* mutations detected in the tissue biopsies were observed in the non-purified ctDNA samples (Figure 6). By contrast, analysis of the 42 purified ctDNA samples revealed 14 samples harboring oncogenic mutations, with oncogenic *KRAS* mutations being the most prominent (*n* = 10), followed by oncogenic *EGFR* mutations (*n* = 4). Of the total 17 samples with an oncogenic *KRAS* mutation, only in six samples (35%) the mutation was detectable in all three approaches (tissue biopsy, non-purified ctDNA and purified ctDNA). For two samples (12%) *KRAS* mutations were observed in tissue biopsy and purified ctDNA, whereas in six samples (35%) *KRAS* mutation was detected only in the corresponding tissue biopsy. Further, we observed six samples with an oncogenic mutation in *EGFR*, of which three samples (50%) showed the same mutation in tissue biopsy and purified ctDNA. Moreover, four oncogenic mutations in *EGFR* (*n* = 1) or *KRAS* (*n* = 3) were exclusively identified in purified ctDNA, while no oncogenic mutations were seen only in non-purified ctDNA or in a tissue biopsy and non-purified sample. When comparing the results obtained from the tissue biopsy with those of our non-purified ctDNA samples, we obtained an overall concordance of 70%, while for the purified ctDNA samples an overall concordance of 71% (Figure 5A) was achieved. Regarding the mutation specific concordance, we observed an increase from 32% to 79% after purification. In most of the cases for which we detected the oncogenic mutations in all three samples, the purification led to a distinct increase in the variant allele frequencies (VAFs), except for samples #13 and #34. For those cases with a VAF of 1% in the purified samples, the mutation in the non-purified sample was not detected (Figure 7), leading to a sensitivity of 58% for the purified or 33% for the non-purified ctDNA samples. Collectively, our results show that our purification protocol is a helpful tool to rescue inappropriately handled ctDNA with lower quality. With material costs below €10 per samples, as only magnetic beads, ethanol and Agilent Bio-Analyzer chip are required, the purification is also a cost-effective method to rescue ctDNA from blood samples with clinical relevance.

## 3. Discussion

**Figure 8:**
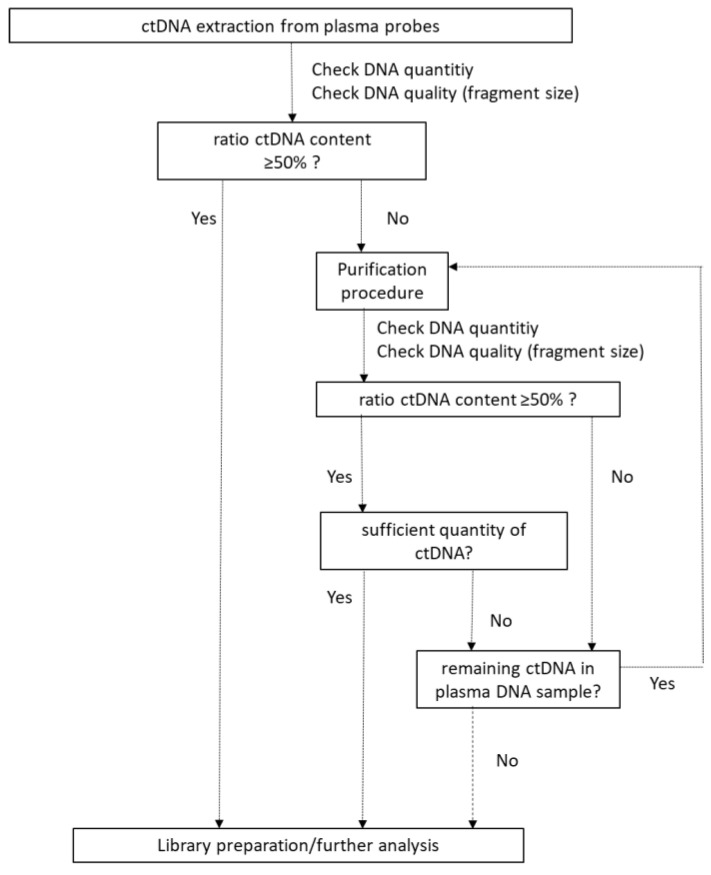
Decision tree for the application of the plasma DNA purification protocol. For low quality ctDNA a purification procedure is recommended. A purification procedure can be repeated until the best possible result is achieved.

Tumor heterogeneity leads to variations in the mutational spectrum at different tumor sites [6]. While tissue biopsy being the gold standard in tumor genotyping, inaccessible tumor sites, highly metastatic tumors as well as tissue biopsies with insufficient tumor cell content due to fine needle biopsies display major problems in daily routine [22]. For those cases molecular genotyping may not be feasible at all [23]. Therefore, analysis of ctDNA derived from liquid biopsies is thought to unravel the aggregation of all mutations at a metastatic tumor, thus, being an alternative to conventional biopsies [24]. However, several studies had demonstrated that liquid biopsy processing has a high impact on further approaches [13,25,26]. For blood stored in tubes containing EDTA longer than 24 h prior plasma isolation, contamination of ctDNA with cellular DNA from dying immune cells was observed [13]. As this is one main issue in archived blood samples with generally improper handling, improvement of these methods are mandatory to rescue these inappropriately handled samples. The impact of the improper handling of blood and plasma samples is also evident in our proof-of-concept study. For instance, overall plasma DNA concentrations from liquid biopsies stored for four days harbor distinctively more DNA than those stored for one or two days (Figure 2). Furthermore, also the percentage of contaminating longer DNA fragments increased with the delay in plasma preparation (Figure 3). This is in line with a study from Parpart-Li et al., which demonstrated that storage temperature plays an important role in ctDNA quality, hence, storage temperature at 4 °C delays contamination with cellular DNA of up to 3 days [27]. Further, Wong et al. observed not only the processing time but also shipping and storage temperature as well as physical shock to have an effect on cell integrity in blood samples used for a prenatal diagnosis [28]. Other studies suggested that utilization of Cell-Free DNA BCT tubes (Streck, La Vista, Nebraska) or PaxGene tubes (Qiagen, Hilden, Germany) significantly decrease contamination of the ctDNA with cellular DNA [13,20] by preventing cell damage due to cell-preserving reagents. However, even with the usage of such specific sample tubes, lysis of peripheral blood lymphocytes (PBLs) is not completely abolished [21]. The data presented here clearly show that our purification procedure is capable of rescuing such contaminated plasma DNA samples. For instance, we enriched ctDNA from 31% to a mean purity of 77%, which could be subsequently used for next-generation sequencing approaches leading to an increase in the mutation specific concordance from 32% to 79% after purification. Further, when comparing the tested non-purified and purified ctDNA samples with oncogenic mutations we demonstrated an increase in sensitivity from 33% with non-purified to 58% with purified samples. However, despite successful enrichment of ctDNA, not all oncogenic mutations already seen in tissue biopsy were observed in purified plasma DNA samples. The reason for this deficiency remains unclear, but it might be explained by a very low abundance of the corresponding ctDNA molecules in the blood or very small amounts of plasma DNA samples. By contrast, the additional four oncogenic mutations exclusively seen with the purified ctDNA are most likely derived from other tumor sites in the patients and therefore underscore the general potential of liquid biopsies in analyzing tumor heterogeneity, as one single biopsy with a small sample size cannot reveal the whole mutational landscape of a tumor. Therefore, our purification procedure is able to improve the outcome of inappropriately handled plasma samples for molecular genotyping in a time and cost-effective manner. However, as we observed a partial loss of plasma DNA during the purification procedure, we recommend using the purification procedure only for cases with less than 50% in the fragment size of ctDNA (Figure 8, Table S2).

Taken together, we presented here a useful procedure to rescue non-informative ctDNA samples.

## 4. Materials and Methods 

### 4.1. Patients

From May 2014 to July 2017, 600 patients with NSCLC were enrolled in the LuCaBiO study (Lung Cancer and Biological Outcome, NCT02613637). All patients participating were required to meet the criteria: (a) the patients had to be diagnosed with NSCLC adenocarcinoma, (b) all patients accepted biopsy with sufficient tumor tissue to detect genetic mutations and (c) patients provided sufficient plasma for genetic detection. Finally, 51 patients meeting the criteria were selected for ctDNA analysis, 37% patients were female and mean age was 63.3 years. For all patients, analyses of tumor tissue were performed according to the IASLC UICC TNM (7th edition) classification. Analyses include the morphology of tumor cells, immunophenotyping and molecular genetic studies. Biobanking of patient tumor tissue was organized in the local pathology units. The study was approved by the hospital’s ethics committee (ethic code 371/13) and all patients gave signed informed consent.

### 4.2. Human Tissue Biopsy

Human tissue biopsies used in the current study were collected and stored by the Institute of Pathology of the University Medical Centre Ulm. Tissue biopsies were fixed in formalin and further paraffin embedded and stored at room temperature until use. Pathologists assessed all samples before use. Extraction of FFPE DNA was performed using the Qiagen FFPE DNA Mini Kit (QIAGEN, Hilden, Germany) according to the manufacturer’s instructions. For each patient we used one to three tissue sections (5 µm) for DNA extraction depending on the tumor size. DNA was recovered in 25 µL of elution buffer and stored at −20 °C until further use.

### 4.3. Plasma Collection

Whole venous blood (7.5 mL) was collected in K2EDTA tubes (Sarstedt, Nümbrecht, Germany) by peripheral blood withdrawal. Plasma separation was carried out within up to 7 days after collection. Whole blood was centrifuged for 10 min (2500× *g* at room temperature) and the plasma fraction was recovered in two fresh 2 mL tubes for immediate storage at −80 °C until ctDNA isolation.

### 4.4. Extraction of Circulating Cell-Free DNA

Circulating cell-free DNA was extracted from plasma using the QIAamp Circulating Nucleic Acid Kit (QIAGEN, Hilden, Germany) according to the manufacturer’s instructions. For each patient we used 2 mL of plasma for ctDNA extraction and recovered ctDNA in 25 μL of elution buffer. DNA was stored at −20 °C until further use.

### 4.5. Quantification of FFPE and Cell-Free DNA

The total amount of DNA was determined by fluorometric measurement using Qubit 3.0 Fluorometer (ThermoFisher Scientific, Waltham, MA, USA). We used 1 μL of DNA eluate gained through the QIAamp Circulating Nucleic Acid Kit (Hilden, Germany) and measured the concentration using the Qubit dsDNA HS Assay Kit (Life Technologies, Carlsbad, CA, USA) according to the manufacturer’s instructions. Fragment size of DNA was determined by electrophoresis using Agilent Bio-Analyzer 2100. We used 1 µL of DNA eluate and measured the fragment size distribution using the Agilent High Sensitivity Kit (Agilent Technologies, Santa Clara, CA, USA) according to the manufacturer’s instructions. 

### 4.6. DNA Purification Procedure

DNA size selection was done using Agencourt AMPure XP magnetic beads (Beckman Coulter, Brea, CA, USA) to purify ctDNA with a fragment length of 90–250 bp, according to the manufacturer’s instructions. 25 µL of plasma DNA was mixed with 1.2× AMPure XP magnetic beads and incubated for 5 min at room temperature. After 5 min of incubation at the magnetic rack, the supernatant was convicted into a new tube. Beads containing longer fragments (≥1000 bp) were washed twice with 80% ethanol, air-dried and longer fragments were eluted with 20 µL RNase free water. Meanwhile, 0.6× AMPure XP beads were added to the supernatant and incubated for 5 min at room temperature. After additional incubation for 5 min on the magnetic rack, the supernatant was discarded and beads were washed twice with 80% ethanol and air-dried. Purified ctDNA was eluted with 20 µL RNase free water. The quality of purified ctDNA was checked using Agilent Bio-Analyzer High Sensitivity DNA Kit (Santa Clara, CA, USA).

### 4.7. Next Generation Sequencing and Data Analysis

FFPE tissue DNA, non-purified and purified ctDNA were further used for amplicon sequencing. For each library up to 20 ng DNA/ctDNA were used depending on the amount of available DNA/ctDNA (min = 1.1 ng; max = 20 ng; mean =10.9 ng). The library was prepared using the Tumor Actionable Mutations Gene Read kit (Qiagen, Hilden, Germany), which allows the sequencing of *BRAF, EGFR, IDH1, IDH2, KIT, KRAS, NRAS* and *PDGFRA* gene segments. Prepared libraries were sequenced by targeted next-generation sequencing on a MiSeq platform (Illumina, San Diego, CA, USA) with paired-end 150-base pair reads (approximately 5000× coverage). Fastq files were uploaded to Qiagen CLC Biomedical Workbench V5.2 (Hilden, Germany), samples were analyzed using a ready-to-use workflow “Identify and Add Variants” and reads were mapped against Human Genome Build 19 (hg19) as the reference. Additionally, the Integrative Genomics Viewer (IGV) was used to visualize variants.

### 4.8. Statistical Analysis

For the calculation of the concordance, the same mutations detected in both matched FFPE tumor and ctDNA samples were classified as true positives; true negatives were identified as those where both matched tumor and ctDNA samples had no mutations; mutations identified in ctDNA, which were not found in tumor tissue DNA were classified as false positives and mutations identified in tumor tissue DNA but not in ctDNA were classified as false negatives. The overall concordance rate was defined as the ratio of the sum of the number of true positives and true negatives to the total enrolled patients. Concordance between tissue mutated samples and purified ctDNA was calculated as the ratio of the number of mutations in the tissue biopsy to purified ctDNA. Sensitivity rate was defined as the ratio of the true positives to the sum of true positives and false negatives. Significance was determined using a Student’s *t* test, with a *p*-value of <0.05 showing statistical significance.

## 5. Conclusions

This procedure is easy to perform, time and cost effective and leads to purified ctDNA, which is sufficient to detect oncogenic mutations in NGS approaches. Moreover, the efficacy of our purification procedure is independent of the delay in plasma DNA preparation and degree of contamination with longer DNA fragments making it a valuable technical improvement in all laboratories handling liquid biopsy samples.

## Figures and Tables

**Table 1:** Characteristics of the cohort.

Patients	51
age	63.3 ± 9.7
	*N* (%)
male	32 (63)
female	19 (37)
	*N* (%)
adenocarcinoma	45 (88)
squamous cell carcinoma	6 (12)
metastatic sites	*N* (%)
0	9 (18)
1	16 (31)
2	10 (20)
>2	12 (23)
n/a	4 (8)
UICC7 stage	*N* (%)
IA	2 (4)
IIA	1 (2)
IIB	1 (2)
IIIA	4 (8)
IIIB	4 (8)
IV	38 (74)
n/a	1 (2)

## Supplementary Materials

The following are available online at https://www.mdpi.com/2072-6694/12/7/1917/s1, Table S1: percentage of ctDNA content, Table S2: ctDNA content in ng/mL plasma.
